# Patterns of Pneumatization of the Posterior Nasal Roof

**DOI:** 10.3390/tomography8010026

**Published:** 2022-02-02

**Authors:** Alexandru Nicolae Mureșan, Mugurel Constantin Rusu, Petrinel Mugurel Rădoi, Corneliu Toader

**Affiliations:** 1 Division of Anatomy, Department 1, Faculty of Dental Medicine, “Carol Davila” University of Medicine and Pharmacy, RO-020021 Bucharest, Romania; alexandru-nicolae.muresan@drd.umfcd.ro; 2 Research Department, “Dr. Carol Davila” Central Military Emergency Hospital, RO-010825 Bucharest, Romania; 3 Division of Neurosurgery, Department 6—Clinical Neurosciences, Faculty of Medicine, “Carol Davila” University of Medicine and Pharmacy, RO-020021 Bucharest, Romania; corneliu.toader@umfcd.ro; 4 Clinic of Neurosurgery, “Dr. Bagdasar-Arseni” Emergency Clinical Hospital, RO-041915 Bucharest, Romania

**Keywords:** cribriform plate, ethmoid bone, sphenoid sinus, anterior cranial fossa, paranasal sinuses, pneumatization, nasal roof, skull base

## Abstract

*(1) Background:* For good surgical performance, sound knowledge of anatomy is required. Although the ethmoid air cells and sphenoid sinuses are subject to a high degree of variation, their possible extensions above the nasal fossa at the posterior end of the cribriform plate of the ethmoid bone (CPEB) were seemingly overlooked. *(2) Methods:* We retrospectively studied 162 case files from 55 male and 107 female cases, with ages varying from 42 to 80, which were scanned using Cone Beam Computed Tomography. *(3) Results:* In 56.17% of cases, an unpneumatized CPEB (type I) was found. Nasal roof recesses of ethmoidal origin (type II) were found at the posterior end of the CPEB in 20.37% of cases. Different types of sphenoidal pneumatizations of the posterior end of the CPEB (type III) were found in 22.83% of the cases. Onodi cells projected nasal roof recesses (type IV) in only 10 cases. In all types, nasal roof recesses were found either above the CPEB or within/underneath it. Moreover, such nasal roof recesses were found to be either unilateral, extended contralaterally, or bilateral. *(4) Conclusions:* As such recesses of the posterior CPEB, previously overlooked, belong to the posterior rhinobase, they should be carefully documented preoperatively to avoid unwanted surgical damage to the olfactory bulb or CSF fistula.

## 1. Introduction

The preoperative diagnostic tools currently available allow for a great therapeutic effect and minor iatrogenic injuries [1]. The basis for minimizing damage in the surgical field is a comprehensive knowledge of microsurgical anatomy [1].

The floor of the anterior cranial fossa (ACF) is mainly formed in its median region by the cribriform plate (sieve plate) of the ethmoid bone (CPEB) fitted into the ethmoidal notch of the frontal bone [1]. On it lies the olfactory bulb. The CPEB is located at the junction of the frontal sinuses, ACF, and nasal fossae and is therefore equally relevant for ENT surgeons and neurosurgeons [2]. The nasal fossae are separated by the nasal septum in the midline [3]. The CPEB shows remarkable shape variations [4]. Anatomic variations of the ethmoid air cells and nasal fossae are common and of clinical relevance to endoscopic surgical management in this area [5]. Sphenoid sinus masses can extend to the ethmoidal labyrinth and break the CPEB [6]. Moreover, as the CPEB is a thin portion of the skull base, it is susceptible to lesions [7,8]. Fractures in the anterior fossa usually cross the CPEB [9]. The CPEB could also be injured during a nasal swab, such as in the procedure for COVID-19 testing [10]. Rarely occurring arterial variants above the CPEB, such as the nasal branch of the persisting primitive olfactory artery [11], indicate that the common anatomical details on that plate are not exclusive.

Laterally, the cribriform plate joins the ethmoidal labyrinth. Posteriorly, it articulates with the sphenoid body, building a slight upward bony stair [1]. Although the sphenoid body is pneumatized, as is the ethmoidal labyrinth, the anatomic possibilities of pneumatic extensions of these sinuses above or within the CPEB have not been reported in previous studies [12,13]. We aimed at investigating the pneumatic recesses of the nasal roof by using retrospective Cone Beam Computed Tomography (CBCT) scans. This is because the anatomical details are better displayed in CBCT than in spiral CT scans.

## 2. Materials and Methods

There were 175 retrospectively documented consecutive DICOM files from 66 male and 109 female patients aged 38 to 83, which were scanned in Cone Beam Computed Tomography (CBCT). Exclusion criteria were pathological changes in the investigated area, surgical procedures involving the anterior skull base or nasal cavity, and blurred or incomplete CBCT scans of the skull base. Inclusion criteria were complete anatomical details of the nasal cavity and skull base and good-quality CBCT scans. Therefore, of the 175 cases, 13 patients, 11 male and 2 female, were excluded due to an insufficient vertical course of the scanner, which did not allow visualisation of the CPEB. Therefore, 162 files from 55 male and 107 female cases, with ages varying from 42 to 80, were kept for the study.

The subjects had been scanned using an iCat CBCT machine (Imaging Sciences International [Hatfield, PA, USA]) with the following settings: resolution 0.250, field of view 130, and image matrix size 640 × 640. The CBCT data were analysed using Planmeca Romexis Viewer 3.5.0.R software, as in previous studies [14,15,16]. The patients gave written informed consent for their data to be used if anonymized. The study was approved (no. 456/4 May 2021) by the responsible authorities (the second affiliation of the first author).

**Figure 1:**
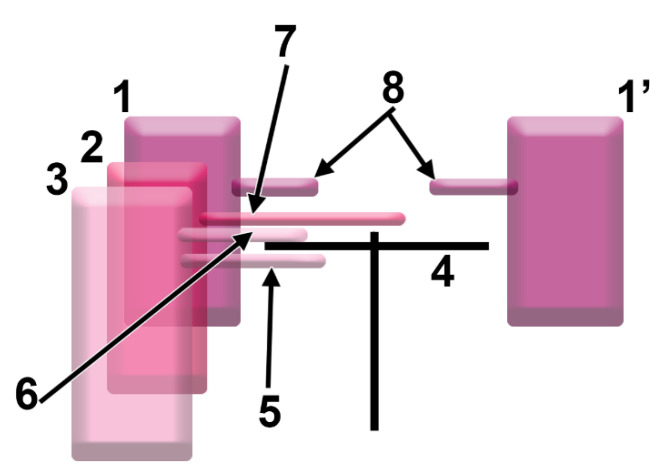
Diagram of the pneumatic patterns at the level of the posterior end of the cribriform plate of the ethmoid bone (CPEB). Coronal view. The sphenoidal sinus (**1**—right side, **1’**—left side), sphenoethmoidal or Onodi cells (**2**), and posterior ethmoidal cells (**3**) could potentially project recesses above or beneath the CPEB (**4**). These recesses could be placed beneath the CPEB (**5**, subtype “b”), or above it (**6**–**8**, subtypes “a”). These recesses could be unilateral (**5**,**6**, pattern “1”), extended contralaterally (**7**, pattern “2”), or bilateral (**8**, pattern “3”).

Different patterns were defined (Figure 1) to be further documented. These were grouped into four types: type I: non-pneumatized posterior end of the CPEB; type II: ethmoidal origin of the pneumatizations of the CPEB; type III: sphenoidal origin of the pneumatizations of the CPEB; and type IV: pneumatization of the CPEB extended from Onodi cells (sphenoethmoidal cells). Each pneumatized type was subdivided into two subtypes, depending on the topography of the respective pneumatization, either protruding into the anterior cranial fossa (intracranial “a” subtypes) or located within and/or immediately beneath the CPEB (intranasal “b” subtypes). Moreover, for each subtype, the following three patterns were observed: “1”: unilateral nasal roof pneumatization; “2”: contralaterally extended nasal roof pneumatization; and “3”: bilateral nasal roof pneumatizations with similar origins.

For each documented DICOM file, coronal, sagittal and axial slices through the CPEB were observed. If no pneumatization of the posterior end of the CPEB was found, we recorded the case as “type I”. Pneumatizations of ethmoidal origin were recorded as “type II”. Those of sphenoidal origin were recorded as “type III”, and those originating from an Onodi cell were classified as “type IV”. Pneumatizations above the CPEB were recorded as subtypes “a”, and those within or beneath the CPEB were recorded as subtypes “b”. For each recorded pneumatic type and subtype, we checked on whether the coronal and axial slices are unilateral (pattern “1”) or extend contralaterally (pattern “2”) or are bilateral (pattern “3”). Relevant anatomical features were exported as image files.

## 3. Results

Different patterns of pneumatization of the posterior nasal roof were recorded, except for types IIb2 (contralaterally extended nasal roof recess of the ethmoid beneath the CPEB) and IVa (Onodi cells extending above the CPEB).

**Figure 2:**
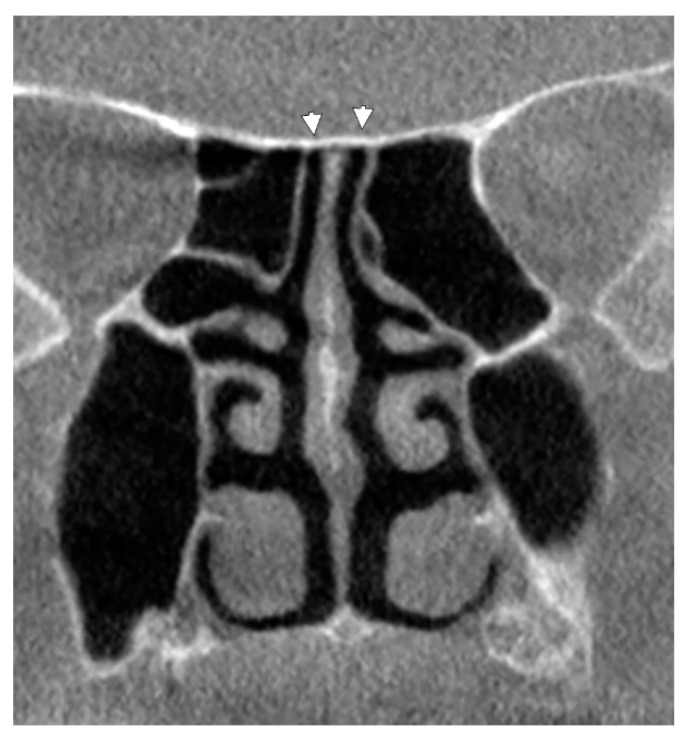
Coronal slice through the posterior end of the cribriform plate. Unpneumatized (arrowheads) nasal roof (type I).

Type 1 of the CPEB, therefore, unpneumatized CPEB, was found in 91/162 cases (56.17%), 28 male and 63 female (Figure 2). Therefore, in 43.83% of cases, different pneumatizations were either added over (the “a” subtypes) or were replacing (the “b” subtypes) the posterior end of the CPEB.

### 3.1. Ethmoidal Origin of Nasal Roof Pneumatizations

Nasal roof recesses of ethmoidal origin were found at the posterior end of the CPEB in 33/162 cases (20.37%), uncombined or in combinations (Table 1).

**Figure 3:**
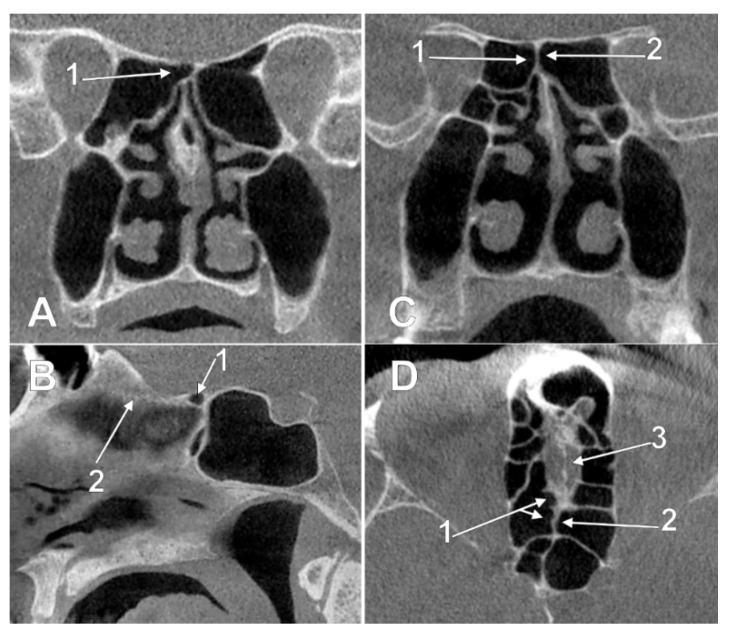
Left panel. Intracranial ethmoidal pneumatization of the posterior nasal roof, derived from a right posterior ethmoidal cell (type IIa2), (**A**) coronal slice, (**B**) sagittal slice. 1.nasal roof recess of the right posterior ethmoid air cell; 2.cribriform plate. Right panel. Bilateral intracranial ethmoidal pneumatization above the posterior end of the cribriform plate (type IIa3), (**C**) coronal slice, (**D**) axial slice. 1. nasal roof recess of the right posterior ethmoid air cell; 2. nasal roof recess of the left posterior ethmoid air cell; 3. cribriform plate.

**Figure 4:**
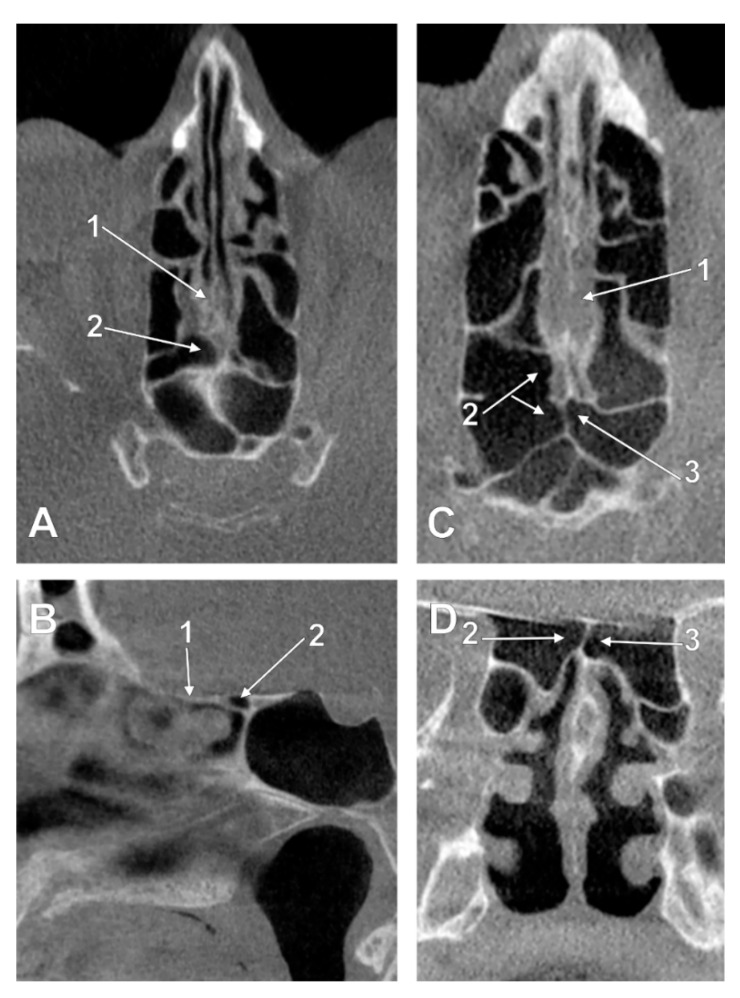
Left panel. Intranasal unilateral ethmoidal pneumatization of the cribriform plate (type IIb1). (**A**) axial slice. (**B**) sagittal slice, right side. 1. cribriform plate; 2. unilateral ethmoidal recess within the posterior end of the cribriform plate. Right panel. Intranasal bilateral ethmoidal pneumatizations of the cribriform plate (type IIb3). (**C**) axial slice. (**D**) coronal slice. 1. cribriform plate; 2. nasal roof recess of a right posterior ethmoid cell; 3. nasal roof recess of a left posterior ethmoid cell.

In three cases (1.85%), one male and two female, the uncombined type IIa1 was found—unilateral ethmoidal pneumatizations extended above the CPEB. The uncombined IIa2 type was found in two cases (1.23%): male and female (Figure 3A,B). The uncombined IIa3 type was found in a male case (0.61%) (Figure 3C,D). The uncombined type IIb1, unilateral ethmoidal pneumatization within or beneath the CPEB, was found in 8/162 cases (6.06%) (Figure 4A,B). In 12/162 cases (7.4%), three male and nine female, the uncombined type IIb3 was found (Figure 4C,D), with the bilateral intranasal ethmoidal pneumatizations replacing the posterior end of the CPEB.

**Figure 5:**
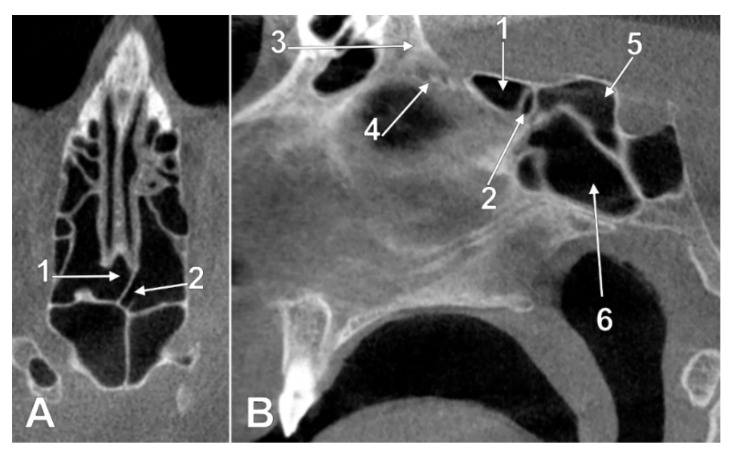
Intracranial ethmoidal pneumatizations of the cribriform plate. Combination of the IIa1 and IIa2 types. (**A**) axial slice; (**B**) sagittal slice. 1. contralaterally extended nasal roof recess of a right posterior ethmoid cell; 2. unilateral nasal roof recess from a left posterior ethmoid cell; 3. crista galli; 4. cribriform plate; 5. right sphenoidal sinus; 6. left sphenoidal sinus.

**Figure 6:**
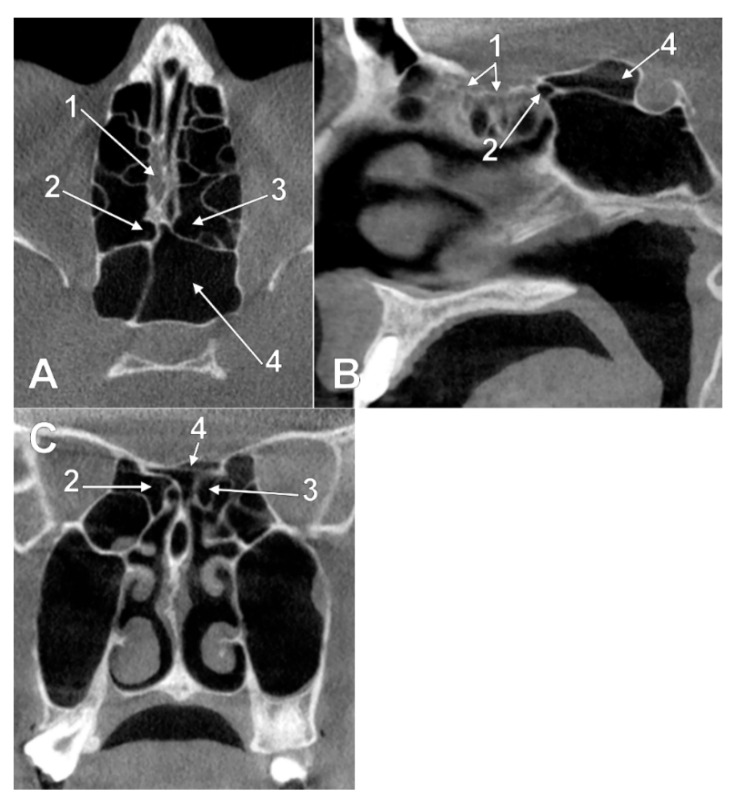
Intracranial ethmoidal and sphenoidal pneumatizations of the nasal fossa roof deriving from ipsilateral ethmoid air cells and the left sphenoidal sinus (combined types IIa3 and IIIa2). (**A**) axial slice; (**B**) sagittal slice; (**C**) coronal slice. 1. cribriform plate; 2. right posterior ethmoid air cell extending above the right nasal fossa; 3. left posterior ethmoid air cell extending above the left nasal fossa; 4. left sphenoidal sinus extended above the nasal roof ethmoidal extensions.

In two male cases (2/162, 1.23%), the combination of IIa1 and IIa2 types (Figure 5) was found. In a female case (1/162, 0.61%), the combination of IIa3 and IIIa2 types was found; therefore, the bilateral ethmoidal pneumatization above the CPEB was supplied from both ethmoidal labyrinths to which a contralaterally extended recess of the left sphenoidal sinus was added (Figure 6).

**Figure 7:**
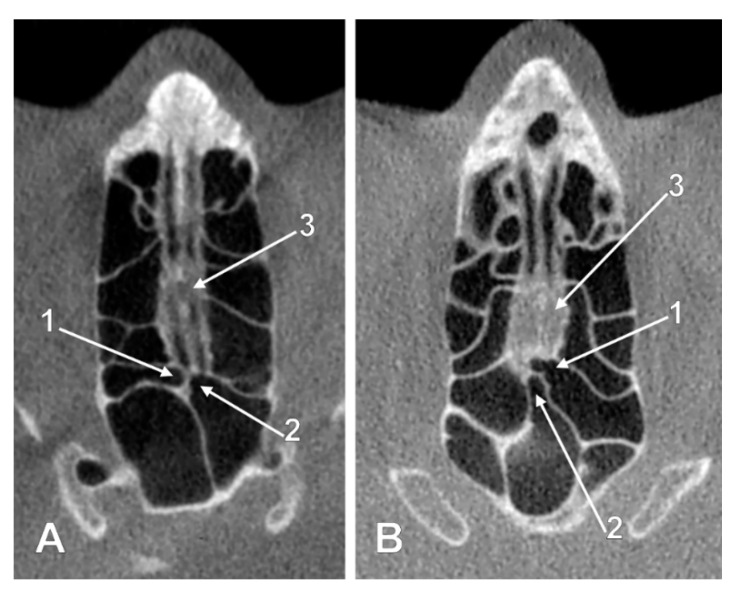
Combinations of pneumatic types. (**A**) Axial slice of an IIa1 + IIIa1 combined type. 1. unilateral nasal roof recess of a right posterior ethmoid cell; 2. unilateral nasal roof recess of the opposite sphenoidal sinus; 3. cribriform plate. (**B**) Axial slice of an IIa1 + IIIa2 combined type. 1. unilateral nasal roof recess of a left posterior ethmoid cell; 2. median recess of the left sphenoidal sinus above both nasal fossae; 3. cribriform plate.

**Figure 8:**
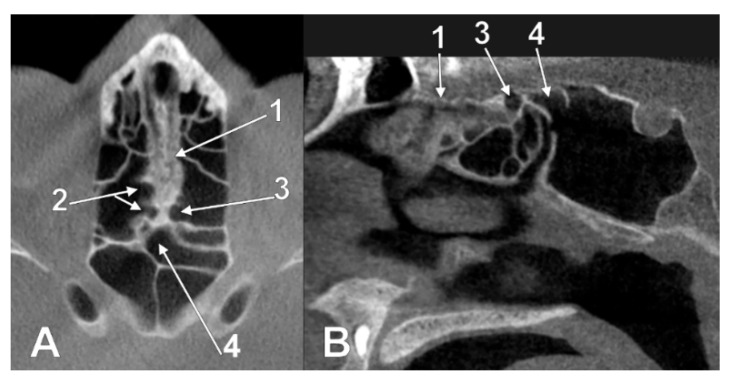
Combination of ethmoidal and sphenoidal pneumatizations of the posterior nasal roof, IIb3 + IIIb2 types. (**A**) axial slice; (**B**) sagittal slice. 1. cribriform plate; 2. right ethmoidal recesses of the posterior nasal roof; 3. left ethmoidal recess of the nasal roof; 4. left sphenoidal recess of the nasal roof extended contralaterally.

**Figure 9:**
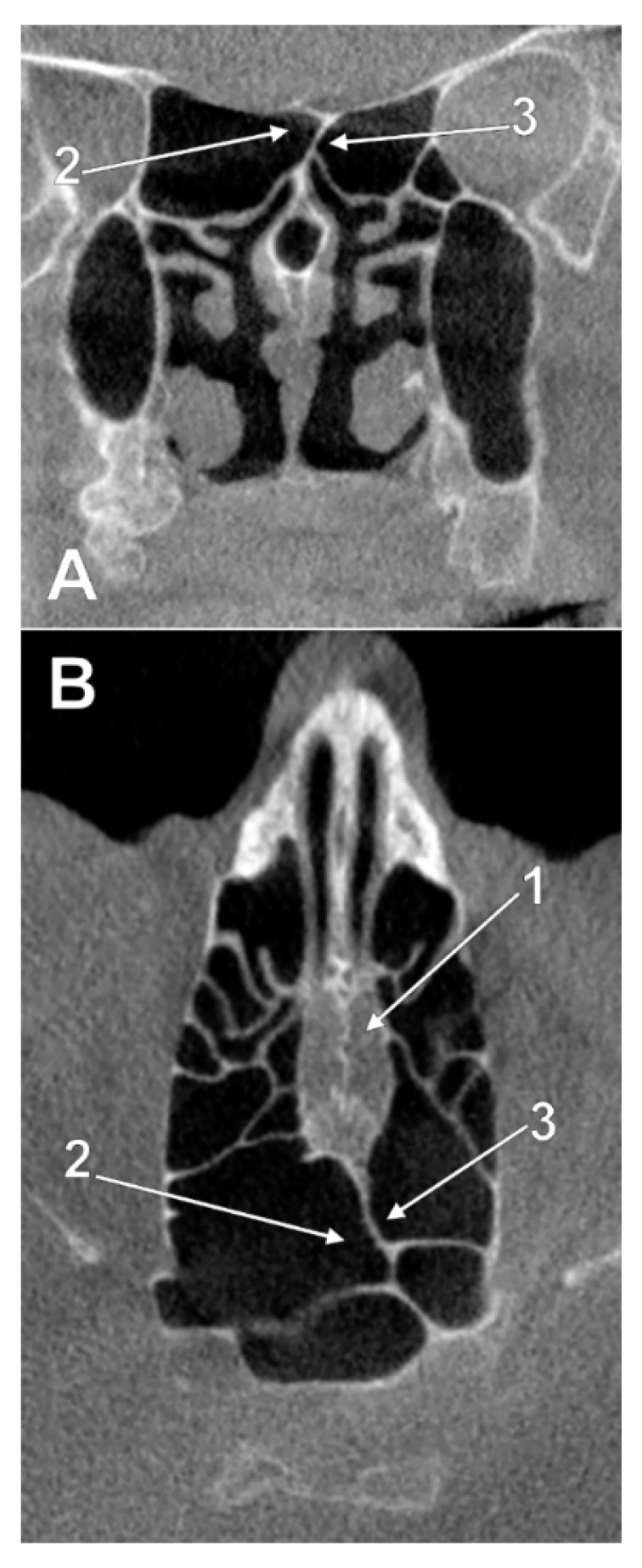
Two-layered intranasal pneumatization of the left nasal roof (combined types IIb1 and IVb2). (**A**) coronal slice; (**B**) axial slice. 1.cribriform plate; 2.right Onodi cell extending over both nasal fossae; 3.left posterior ethmoid air cell extended above the ipsilateral nasal fossa, but under the recess of the contralateral Onodi cell.

In a different female case (0.61%), the combined types IIa1 and IIIa1 were found, i.e., unilateral ethmoidal and sphenoidal pneumatizations were found to be above the posterior end of the CPEB (Figure 7A). Another female case (0.61%) had a combination of the IIa1 and IIIa2 types, in which, there was unilateral ethmoidal pneumatization of the CPEB but contralaterally extended sphenoidal pneumatization of the nasal roof (Figure 7B). In one male case (0.61%), the combination of the IIb3 and IIIb2 types was found whereby the bilateral ethmoidal pneumatization beneath the CPEB was supplied from both ethmoidal labyrinths, to which a contralaterally extended recess of the left sphenoidal sinus was added (Figure 8). In another male case (0.61%), the combination IIb1 + IVb2 was found, i.e., on the left side, a posterior ethmoid air cell extended beneath the CPEB and a right Onodi cell extended contralaterally beneath the CPEB (Figure 9).

### 3.2. Sphenoidal Origin of Nasal Roof Pneumatization

**Figure 10:**
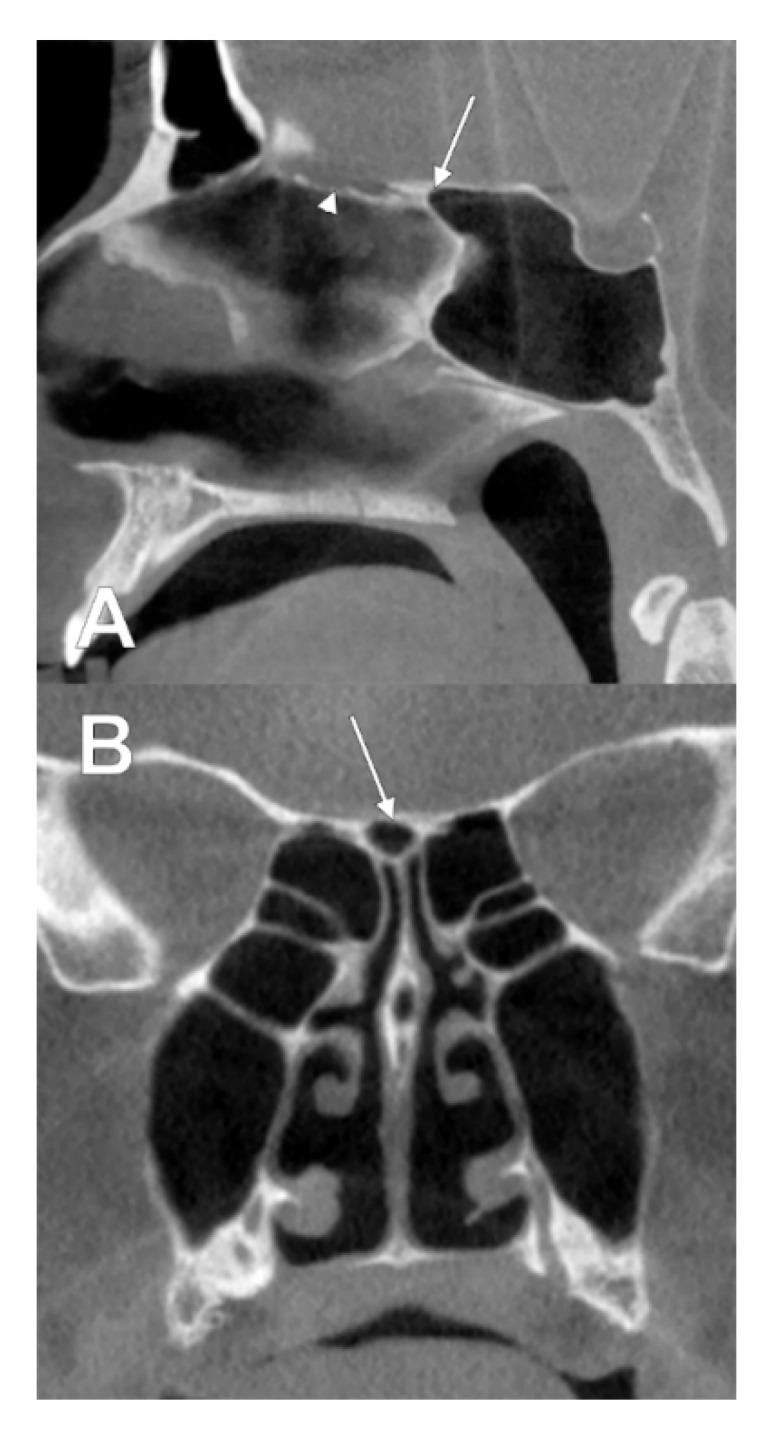
Intracranial sphenoidal pneumatization of the posterior nasal roof, derived from the right sphenoidal sinus (type IIIa2). (**A**) sagittal slice; (**B**) coronal slice. The superior median extension of the right sphenoidal sinus (**A**,**B**, arrow) replaces the posterior end of the cribriform plate (**A**, arrowhead).

Different types of sphenoidal pneumatizations of the posterior end of the CPEB were found in 37/162 cases (22.83%), uncombined or in combinations (Table 1). Unilateral sphenoidal pneumatization above the CPEB (uncombined type IIIa1) was found in three cases (1.85%), one male and two female. The uncombined type IIIa2, a contralaterally extended sphenoidal recess above the CPEB, was found in 5/162 cases (3.08%), one male and four female (Figure 10). The uncombined type IIIa3, i.e., bilateral sphenoidal recesses above the CPEB, was found in just one male case (1.23%). The uncombined type IIIb1 was found in 14/162 cases (8.64%), six male and eight female. The uncombined IIIb2 type, of contralaterally extended sphenoidal recesses beneath the CPEB, was found in 5/162 cases (3.08%), three male and two female. Type IIIb3, i.e., bilateral sphenoidal recesses within or beneath the CPEB, was found in just 2/162 male cases (1.23%).

In three cases (1.85%), the combination IIIb1 + IVb1 was found; therefore, sphenoidal and sphenoethmoidal unilateral extensions located beneath the CPEB.

### 3.3. Sphenoethmoidal (Onodi Cell) Origin of Nasal Roof Pneumatizations

**Figure 11:**
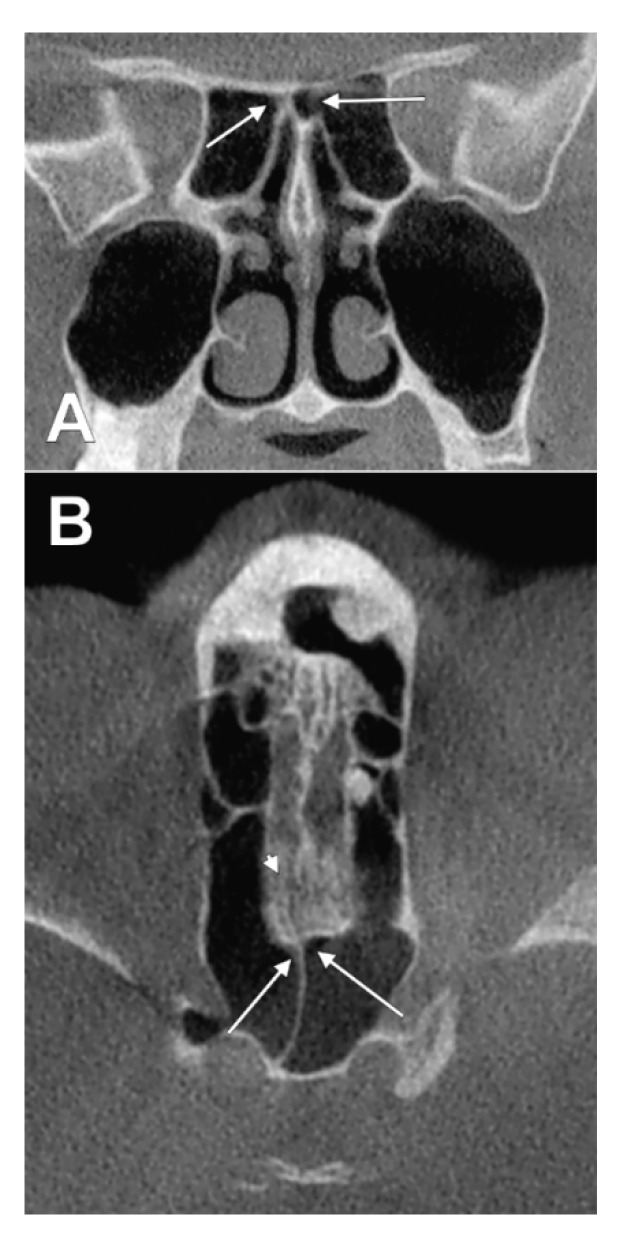
Intranasal bilateral pneumatization of the cribriform plate derived (arrows) from large Onodi cells (type IVb3). (**A**) coronal slice. (**B**) axial slice. The arrowhead in B indicates the cribriform plate.

Type IVb (recesses beneath the CPEB extended from Onodi cells) was found in 10 cases. In four of these, combinations of types already described in this section were found. Therefore, in just 6/162 cases (3.7%), uncombined IVb types were found. The uncombined type IVb1, a unilateral extension of an Onodi cell within or beneath the CPEB, was found in 2/162 cases (1.23%). In 3/162 cases (1.85%), the uncombined type IVb2 was found. In just one female case (0.61%), the uncombined type IVb3—bilateral Onodi cells extending recesses beneath the CPEB—was found (Figure 11).

## 4. Discussion

The present study found that pneumatic spaces near the posterior end of the CPEB could extend the recesses of the CPEB. These recesses could have either ethmoidal, sphenoethmoidal (Onodi), or sphenoidal origins in almost all possible combinations. They could potentially extend to the opposite side, and they could lie above or beneath the CPEB. The present findings contribute to the current specific anatomical knowledge on the paranasal sinuses and anterior cranial fossa floor.

### 4.1. Gore’s Supraseptal Air Cell

Different studies have observed the variational anatomy of the nasal fossa walls [15,17,18,19,20,21,22,23,24,25]. However, these studies did not indicate the variable pneumatization patterns of the nasal roof described here. It has been commonly indicated that the roof of the nasal cavity is formed by the CPEB [26]. Gore (2019) reported a “supraseptal ethmoid sinus cell” that corresponds to the IIa1 type of CPEB pneumatization. The author regarded that finding as the first such case reported in the literature [27]. He noted that “in reviews of hundreds of preoperative maxillofacial CT scans”, that case “had the single example of this particular ethmoid sinus cell” [27]. This is interesting because we found different patterns of pneumatized CPEB in less than 200 cases.

### 4.2. Ethmoidal and Sphenoidal Pneumatizations

In 1939 Van Alyea published a study performed by a dissection of 100 ethmoidal labyrinths [28]. He discussed the fact that ethmoid air cells have a tendency to expand and fill any available space [28]. Such extensions are either common or unusual [28]. Van Alyea termed “postreme cells” those draining above the superior nasal turbinate. Such postreme cells were observed invading the sphenoid sinus or optic canal or expanding medially to reach the nasal septum or the opposite side [28]. However, Van Alyea did not explicitly note the pneumatization of the CPEB by such recesses of the postreme ethmoid air cells.

Van Alyea detailed the anatomy of the sphenoidal sinuses in 1941. He then wrote that the sinus is not limited to the sphenoidal body and that extensions into adjacent bony processes occur regularly [29]. Recesses extending anteriorly from the sphenoidal sinuses are often sufficiently large to occupy a considerable portion of the ethmoid field [29]. The “anterior medial (septal)” recess of the sphenoidal sinus was listed by Van Alyea, but it was not observed that the sinus could also extend above the nasal fossa to override or replace the posterior end of the CPEB, as in the present study. Such anterior supranasal recesses of the sphenoidal sinus are commonly observed in computed tomography scans but are overlooked [30]. A number of other studies have provided important details regarding the numerous anatomic possibilities of the sphenoidal sinuses [8,31,32,33,34,35,36,37,38,39]. However, there is still a substantial amount of research that needs to be conducted on this aspect [31].

Anatomical details of the sphenoid body include several bony projections that could project over the ethmoid anteriorly, including (a) the median, unpaired one is the ethmoidal spine of the sphenoid, commonly triangular and with its tip pointing to the crista galli; (b) the paired lateral processes, if present, are the alae minimae of Luschka [1]. A pneumatized ethmoidal spine would determine a pneumatized posterior nasal roof overriding the CPEB. A broadened anterior septal recess of the sphenoidal sinus could determine adjacent pneumatizations underneath the posterior end of the CPEB.

Onodi cells occur in 3.4–51% of individuals, as documented previously [40]. It is agreed that an Onodi cell is a posterior ethmoid cell that has an intimate relationship with the optic nerve [40,41]. However, when a medial recess of such an Onodi cell roofs the nasal fossa, it will also be closely related to the olfactory bulb above the CPEB.

### 4.3. Clinical Anatomy

A pneumatized posterior nasal fossa roof is a pneumatized roof of the sphenoethmoidal recess. The sphenoid sinus ostium is located either in the posterior wall of this recess or in its lateral wall [42]. Care should be taken when the sphenoid sinus ostium is approached endoscopically to avoid penetrating a pneumatized roof of this recess, that is, a pneumatized posterior end of the CPEB. The CPEB vertical placement is individually variable, and if violated, it could lead to a subsequent encephalocele and cerebrospinal fluid leak [43].

The endoscopic endonasal transcribriform approach is a reliable strategy in the treatment of various anterior skull-base pathologies, such as olfactory groove meningiomas, meningoencephaloceles, esthesioneuroblastomas, schwannomas, and other sinonasal tumours [44]. On a case-by-case basis, it can be converted to a transcranial-endoscopic endonasal transcribriform approach [44]. Such transcribriform approaches should document whether or not the CPEB has an added pneumatic pattern to avoid, if possible, an unnecessary opening of a paranasal pneumatic space.

The lateral lamella of the CPEB is the thinnest part of the olfactory fossa and is at risk during endoscopic surgery [45]. The floor of the olfactory fossa consists of CPEB [13]. Different studies used Keros’ classification [46] of the olfactory fossa depth, which is as follows: in type I, the lateral lamella is short; in type II, the lateral lamella forms the medial part of the ethmoid roof; and in type III, the ethmoid roof is significantly above the CPEB [47]. Another anatomical variable should be documented when the olfactory fossa is evaluated, such as the pneumatic recesses added to the CPEB. Case-by-case evidence would avoid unwanted opening of those sinuses.

Cerebrospinal fluid (CSF) drains through the CPEB in association with olfactory nerves [48]. Therefore, minor anatomic restrictions of the CPEB, such as the added pneumatic recesses, could impede drainage and determine minor increases in resting intracranial pressure [48]. Moreover, when a surgical repair of CPEB [49] is planned, care should be taken to manage the recesses added to the CPEB.

Endoscopic sinus surgery is a standard surgical procedure for chronic rhinosinusitis worldwide [50]. Residual ethmoid cells are incompletely removed air cells and have been thought to be a cause of the recurrence of chronic rhinosinusitis [50]. Therefore, when posterior and post-reme ethmoid air cells are removed, care should be taken if a pneumatized CPEB is approached.

Kainz and Stammberger (1992) discussed the danger areas of the posterior rhinobase and included the Onodi cells and the most posteriorly located cells of the posterior ethmoid in this group [41], the postreme cells of Van Alyea. Onodi cells limit the exposure of the sellar floor, and only after removing these cells is the entire sellar floor exposed so that the tumours can be removed completely [51]. When approaching the sphenoid sinus anteriorly in the endonasal transethmoidal way, the optic nerve can be damaged, depending on the pattern of pneumatization [41]. If the pneumatization of the CPEB is overlooked, the olfactory bulb could also be damaged.

Therefore, the present study contributes to the proper assessment of the important structures in future, when performing endoscopic sinus and skull base surgery, and thus, avoiding further complications. Cribriform plate of the ethmoid bone represents one of the most complex parts of surgical anatomy of human body, so determining its anatomical variations is very necessary to avoid injuring injuries. A surgeon must know the anatomic landmarks very well and this classification of pneumatization of the posterior nasal roof could be of help.

## 5. Conclusions

In conclusion, when the posterior ethmoid and the sphenoid sinus are approached, care should be taken to accurately identify the eventual pneumatizations of the CPEB in order to avoid taking false pathways, which could lead to unwanted surgical events. An anatomical norm, such as the unpneumatized CPEB, seems now just an idealized scientific model, but a useful reference point for clinicians [52].

## Figures and Tables

**Table 1:** Ten from the 162 cases (6.17%) had different combinations of types. Overlap of pneumatisations on the same side was recorded in three from these ten combinations.

Combination of Types	Count	On the Same Side	On Opposite Sides	Males	Females
IIa1 + IIIa1	1		1		1
IIa1 + IIa2	2		2	2	
IIa1 + IIIa2	1	1			1
IIa3 + IIIa2	1	1			1
IIb1 + IVb2	1		1	1	
IIb3 + IIIb2	1		1	1	
IIIb1 + IVb1	3	1	2	2	1
